# Seas of Renewal: Turning Sea Urchin Waste into Polyhydroxynaphtoquinone-Collagen Biomaterials for Regenerative Medicine

**DOI:** 10.1007/s10126-025-10504-2

**Published:** 2025-08-30

**Authors:** Giordana Martinelli, Stefania Marzorati, Margherita Roncoroni, Luciano Magro, Matteo Brilli, Giangiacomo Beretta, Graziano Colombo, Luca Melotti, Anna Carolo, Giulia Zivelonghi, Stefano Farris, Marco Patruno, Raffaella Soave, Mario Italo Trioni, Michela Sugni

**Affiliations:** 1 Department of Environmental Science and Policy, University of Milan, Milan, Italy; 2 Department of Agronomy, Animals and Environment, Natural Resources, University of Padua, Legnaro (Padua), Italy; 3 Department of Biosciences, University of Milan, Milan, Italy; 4 Department of Comparative Biomedicine and Food Science, University of Padua, Legnaro (Padua), Italy; 5 Department of Food, Environmental and Nutritional Sciences, University of Milan, Milan, Italy; 6 Institute of Chemical Science and Technologies “Giulio Natta”, National Research Council, Milan, Italy

**Keywords:** Collagen scaffolds, Polyhydroxynaphtoquinone antioxidants, Sea urchin by-products, Regenerative medicine

## Abstract

Chronic wounds and skin ulcers pose significant challenges to healthcare systems globally, necessitating innovative approaches to accelerate healing processes. Biomaterial-based therapies have emerged as promising solutions for tissue regeneration. This study focuses on valorization of sea urchin waste toward the development and characterization of collagen-based scaffolds added with polyhydroxynaphthoquinone (PHNQ) antioxidants, successfully incorporated into biomaterials at optimal ratio, enhancing scaffold stability and integrity. Water uptake, mechanical properties, and degradation kinetics of the composite scaffolds were evaluated and compared with controls. Biocomposites were also tested for cytotoxicity. Results indicate that composite scaffolds exhibit superior chemical stability and slower degradation rates, attributed to strong interactions between collagen and PHNQs. This aspect was explored also through in silico investigations by means of tight binding molecular dynamics methods. It has been found that a covalent bond forms between the selected collagen representative and one PHNQ. Furthermore, the antioxidant activity of PHNQs was retained in the composite scaffolds, providing additional therapeutic benefits under the perspective application of regenerative medicine. Normal human dermal fibroblasts (NHDF) exposed to the combination of collagen and PHNQs remained viable. Overall, these findings highlight the potential of sea urchin food waste in a valorization chain, offering added value through the production of collagen-based composite scaffolds.

**Supplementary Information:**

The online version contains supplementary material available at 10.1007/s10126-025-10504-2.

## Introduction

Sea urchins are common inhabitants of seas and oceans and, together with their relatives starfishes and sea cucumbers, possess unique biological features, including the presence of mutable collagenous tissue (MCT), *i.e.*, dynamic collagen-based tissues (Candia Carnevali et al. 2024). Some sea urchin species, such as *Paracentrotus lividu*s, are consumed as food, the only edible part being the gonads, a minor fraction of the whole animal body mass (Järbrink et al. 2016; Martinengo et al. 2019). The remaining waste includes the test, the spines and soft tissues such as the peristomal membrane, a well-known MCT surrounding the mouth. The peristomial membrane has been proved to be a valuable source of native fibrillar collagen, still decorated with surface glycosaminoglycans (GAGs), already demonstrated to be useful for biomaterials production (Di Benedetto et al. 2014; Ferrario et al. 2017, 2020). The latter could be prepared in forms of thin films or three-dimensional scaffolds and were successfully in vivo tested for skin regeneration in rat and sheep models (Melotti et al. 2021; Carolo et al. 2023). Indeed, in skin regenerative medicine, there is a critical need for innovative approaches to accelerate wound healing and the development of innovative biomaterial-based therapies holds notable promise (Las Heras et al. 2020). However, biomaterials for tissue regeneration necessitate specific characteristics like biocompatibility, bioactivity, interconnected porosity, mechanical strength, and biodegradability (Lim et al. 2019). Collagen-based biomaterials can satisfy most of these features. While porcine and bovine collagen are commonly used at an industrial level, concerns regarding disease transmission and ethical issues have spurred interest in alternative sources, including marine organisms (Geahchan et al. 2022). In this context, sea urchin collagen may present advantages in terms of safety, sustainability, and mostly in structural-physical properties (Yamada et al. 2014). Indeed, sea urchin collagen biomaterials displayed better mechanical performances when compared to commercial bovine collagen membranes (Ferrario et al. 2017) and the full maintenance of the structural integrity of collagen fibril may provide the biomaterial advantages in terms of hemostatic properties, as the tight fibrillar network facilitates blood coagulation and fibril integrity promotes better platelet activation (Wang et al. 2021). However, compared to standard collagen source as mammals or other marine sources like fish, challenges remain in large-scale extraction of native collagen from sea urchins, due to both process complexity and availability of biomass. These aspects should be carefully addressed in case of future industrial scale-up.

Besides collagen, sea urchins possess peculiar secondary metabolites known as polyhydroxynaphthoquinones (PHNQs), which exhibit potent antioxidant properties (Anderson et al. 1969; Shikov et al. 2018; Rubilar et al. 2021; Melotti et al. 2023). In addition to their radical scavenging ability, PHNQs showed other relevant and potentially exploitable biological activities, such as immune modulation (Shikov et al. 2018; Rubilar et al. (2021), antimicrobial (Brasseur et al. 2017; Hieu et al. 2020), antiviral (Mishchenko et al. 2020), and cardioprotective (Jeong et al. 2014) activities. Several PHNQs have been identified until now, from different sea urchin species (Lebedev et al. 2005; Mischenko et al. 2005; Zhou et al. 2011; Shikov et al. 2018) the most common and best studied being Echinochrome A and Spinochromes A–E (Brasseur et al. 2018). The high pharmaceutical potential of these compounds is demonstrated by the fact that Echinochrome A is the active ingredient of Histochrome, a commercial drug used for cardiological applications in Russia (Rubilar et al.^2021^). PHNQs can be found in different sea urchin tissues, including gonads, coelomic fluid, test and spines, and digestive system (Brasseur et al. 2018) while in the latter, the highest PHNQ concentration can be found, the higher total mass of test and spines can provide quantitatively higher amount of PHNQs. It is important to note that these body compartments actually compose most of the waste discharged after gonad consumption, thus further underlining the possibility to obtain bioactive molecules under the perspective of by-product valorization (Marzorati et al. 2021; Roncoroni et al. 2024).

On the basis of these previous results, the aim of the present study was to develop a “second-generation” composite biomaterial combining fibrillar collagen and PHNQs extracted from the whole sea urchin waste (the peristomial membrane plus the remaining parts) in order to develop a fully ecofriendly device, which allow to maximize waste valorization. The rationale behind was also to combine the beneficial properties of the single components, *i.e*., the structural/mechanical/bioactive feature of MCT-derived collagen and the antioxidant activity of PHNQs, with the aim of further enhancing the therapeutic potential of collagen-based biomaterials and promoting skin wound healing. The so produced PHNQ-collagen composite biomaterials were characterized in terms of structural features, degradation kinetics, mechanical performances, cytotoxicity and antioxidant activity and compared with the simple collagen-based counterpart. Furthermore, a computational approach based on tight binding molecular dynamics simulations was performed.

## Experimental

### Chemicals

Anhydrous sodium sulfate, sodium carbonate, formic acid, ethanol, ethyl acetate, 2,2-azino-bis(3-ethylbenzothiazoline-6-sulfonic acid) (≥ 98%; ABTS), and 6-hydroxy-2,5,7,8-tetramethylchroman-2-carboxylic acid (97%; Trolox^®^) were purchased from Merck (Germany). Acetonitrile, methanol, and water, when used for liquid chromatography, were purchased from Merck (Germany) as ultra-performance liquid chromatography-grade. Ethanol absolute anhydrous was purchased from Carlo Erba (Italy). Trizma^®^ hydrochloride (≥ 99%; Tris–HCl), ethylenediaminetetraacetic acid disodium salt diihydrate (≥ 98.5–101.5%; EDTA), 2-mercaptoethanol (≥ 99%), sodium chloride (NaCl), potassium chloride (KCl), disodium hydrogen phosphate (Na_2_HPO_4_), phosphate buffered saline (PBS), potassium dihydrogen phosphate (KH_2_PO_4_), and collagenase enzyme type I (from *Clostridium histolyticum*) were purchased from Sigma-Aldrich (Germany). Sodium dodecyl sulfate (SDS) was purchased from VWR (Italy).

### Sea Urchin Waste

Sea urchin wastes, belonging to the species identified as *Paracentrotus lividus* (origin: Adriatic Sea), were kindly donated from the restaurants close to the University of Milan after gonad removal. The waste was immediately frozen on site by the restaurant personnel (no cooking or chemical treatment was performed), stored at − 20 °C until collection by the University staff, and immediately transferred to laboratory freezers (− 20 °C) to preserve the target compounds. When necessary, wastes were defrosted and the peristomial membranes were isolated from the test and processed for collagen extraction, while the rest of the waste (*i.e.*, tests, spines, and residual soft tissues such as the digestive tube) was lyophilized to eliminate the residual water (about 40% of the initial biomass). Lyophilization was conducted for 24 h using a 5Pascal Srl (Italy) freeze dryer equipment. The lyophilized material was then ground using a knife mill (Fritsch, Pulverisette 11, Italy), at 10000 rpm for 20 s and the obtained powder was stored at room temperature until use for polyhydroxynaphtoquinone (PHNQ) extraction.

### Collagen extraction

The collagen extraction procedure followed a previously published method (Ferrario et al. (2017, 2020). Briefly, thawed peristomial membranes were rinsed with filtered artificial sea water, weighted, and left overnight at 23 °C under rotating stirring in hypotonic solution (10 mM Tris–HCl, 0.1% ethylenediaminetetraacetic acid tetrasodium salt dehydrate, EDTA, pH 8.0). After several washing cycles in phosphate buffer saline (PBS), samples were immersed overnight in a decellularizing solution (10 mM Tris–HCl, 0.1% sodium dodecyl sulfate, SDS, pH 8.0) at 23 °C. After cleansing thoroughly with PBS to remove SDS, disaggregating solution (0.5 M NaCl, 0.1 M Tris–HCl pH 8.0, 0.1 M β-mercaptoethanol, 0.05 M EDTA) was added and kept at 23 °C for 5 days under rotating stirring. The resulting collagen suspension was filtered on a steel mesh filter, dialyzed against 0.5 M EDTA for 4 h and then against distilled water overnight to completely remove any residue of β-mercaptoethanol. The obtained aqueous collagen suspension was stored at − 80 °C until use. Collagen suspension concentration (mg/mL) was determined by weighting a lyophilized aliquot of 1 mL suspension.

### Collagen Characterization: Amino Acid Profile and SDS Page

Collagen obtained from different extractions was pooled to obtain 50 mL of suspension with a final concentration of 6 mg/mL. This latter was centrifuged at 1500 g for 20 min, the supernatant was removed, and the sample was left at − 80 °C overnight and then freeze-dried (Edwards Pirani 1001). Dried collagen was analyzed at the University of Padua (DAFNAE Department) for amino acid profiling.

Amino acids were analyzed after acid hydrolysis and pre-column derivatization with 6-aminoquinolyl-N-hydroxysuccinimidyl carbamate (AQC), separated by RP-HPLC and analyzed by UV detection following a method adapted from the European Pharmacopoeia. Briefly, for Ala (alanine), Arg (arginine), Asp (aspartatic acid), Glu (glutammic acid), Gly (glycine), His (histidine), Ile (isoleucine), Leu (leucine), Lys (lysine), Met (methionine), Phe (phenylalanine), Pro (proline), Ser (serine), Thr (threonine), Tyr (tyrosine), and Val (valine) determination, the sample was hydrolized with hydrochloride acid (6 M) at 105 °C for 24 h. Cys was determined as sum of cysteine and cystine, after reaction with dithiodipropionic acid, producing a mixed disulfide, which then underwent acid hydrolysis accordingly. After hydrolysis, the samples were neutralized with sodium hydroxide (8 M), adjusted to volume, and filtered at 0.22 µm. Then, the derivatization step was conducted according to the manufacturer’s instructions (Waters, AccQTag Ultra Derivatization Kit). Tryptophan was determined following a method adapted from CD 2000/45/EC using a basic hydrolysis with barium hydroxide at 105 °C for 24 h, and after neutralization and filtration analyzed directly by RP-HPLC. Separation and quantification of amino acids were performed using an Agilent 1260 Infinity HPLC (Agilent Technologies, Santa Clara, CA, USA) equipped with a reversed-phase column C18 (CORTECS C18, 2.7 µm, 2.1 × 150 mm) kept at 45 °C, and with a diode array Detector (Agilent 1260 Series, DAD VL +). The obtained results were expressed in g/100 g, *i.e.*, grams of each amino acid on 100 g of initial material (collagen).

To assess collagen purity (Figure S1), an aliquot of collagen suspension was hydrolyzed with collagenase. Samples of native collagen, hydrolized collagen, and collagenase were separated by SDS-PAGE using 10% stain-free polyacrylamide gels (Bio-Rad Laboratories). Proteins samples were added to an equal volume of 2 × SDS-PAGE reducing sample buffer (60 mM Tris–HCl, pH 6.8, 10% (v/v) glycerol, 2% (w/v) SDS, 0.01% (w/v) bromophenol blue, 5% (w/v) DTT). Samples were then heated at 95 °C for 5 min before being cooled and loaded onto the gel. After electrophoresis (90 V for 30 min and 130 V for 60 min), fluorophore into the gel was activated for 5 min and bands were visualized using the ChemiDoc Touch Imaging System (Bio-Rad Laboratories).

### PHNQ Extraction

The extraction procedure of PHNQs was based on a literature procedure (Powell et al. 2014) optimized in our laboratories (Marzorati et al. 2021). Briefly, lyophilized sea urchin powder from tests and spines was treated dropwise with 6 M formic acid aqueous solution to decompose the calcium carbonate matrix. About 175 mL of formic acid solution was used for 50 g of sea urchin powder. The reaction was kept under stirring for 2 h at room temperature. The obtained suspension was centrifuged at 4000 rpm for 5 min (Eppendorf, Centrifuge 5804 R), and the supernatant was filtered under vacuum with the aid of a Büchner funnel equipped with a cellulose filter. The aqueous solution was then counter-extracted three times with aliquots of 200 mL of fresh ethyl acetate, and the obtained orange/pink organic layers were collected and pooled together. The separated organic phase was further purified by counter-extraction with fresh milli-Q water to remove the residual formic acid and inorganic salts as much as possible, until the aqueous phase conductivity and pH were close to the milli-Q water ones. Anhydrous sodium sulfate was added to remove the residual water in the organic phase and the extract was then dried using a rotary evaporator (37 °C) and finally by a mechanical vacuum pump.

### PHNQ Characterization: Ultrahigh-Performance Liquid Chromatography Coupled to Photodiode Array (UPLC-PDA)

The characterization of PHNQs was performed on Acquity UPLC equipment (Waters corp., MA, USA) using the following conditions: column: ACQUITY UPLC BEH C18 (50 × 2.1 mm, 1.7 um) (Waters, Milford, MA, USA); column temperature: 34 °C; eluents were as follows: A: water + 0.1% formic acid; B: acetonitrile + 0.1% formic acid. The elution gradient was as follows: 0 min: 25% A: 75% B; 20 min: 0% A: 100% B; 28 min: 0% A: 100% B with the flow rate set at 0.3 mL/min. The samples were kept at 20 °C. The injection volume was 2 µL and the analyses were repeated three times. The detector was an Acquity PDA Detector (Waters, Milford, Ma, USA), and the wavelength was set at 445 nm. Data were processed by the use of Empower 3 workstation.

### PHNQ-Collagen Scaffold Production

Thawed collagen suspensions were centrifuged at 1700 g for 5 min and subsequently suspended in a PHNQ-containing 6% (v/v) EtOH/water solution to obtain a 6 mg/mL suspension. Three different PHNQ-collagen suspensions were prepared differing in the w/w proportion of PHNQ to collagen: 1%, 10%, 50%. In parallel, control suspensions (0% PHNQ) were also prepared. The “suitable” (*i*.*e.*, not forming aggregates) suspensions (controls, 1%, 10% PHNQ-collagen) were used to produce 3D scaffolds. To this purpose, 1 mL of each type of suspension was added to a circular silicone rubber mold (*d* = 1 cm), frozen at − 80 °C for 3 h, lyophilized (Edwards Pirani 100), and stored at − 20 °C until use. Some control scaffolds (0% PHNQ) were artificially cross-linked using a 380-nm UV lamp overnight (12 h, Gelaire^®^, 220 V. 50 Hz, 163 W) (coll-UV) to obtain further controls.

### PHNQ-Collagen Scaffold Characterization

- **Ultrastructure (SEM):** The microstructure and ultrastructure (surface, thickness) of composite scaffolds were analyzed by scanning electron microscopy to evaluate the homogeneity of the fibrillar network, and the eventual presence of aggregates. Lyophilized scaffolds were mounted on stubs by means of conductive tape, gold-sputtered with a Leica ACE600 147 sputter coater (Leica Microsystems, Germany), and photographed under a FE-SEM Sigma (ZEISS, Germany) scanning electron microscope (5 kV, working distance WD 15 mm). Fiber diameter and pore size were calculated from SEM images by means of ImageJ software (version 1.54 h) as average of 100 fibers and 15 pores.
- **Attenuated total reflectance Fourier-transform infrared (ATR-FT-IR) spectroscopy:** ATR-FT-IR spectra of collagen-based scaffolds were recorded in the range 3900–400 cm^−1^, using an Alpha spectrometer equipped with an ALPHA’s Platinum single reflection diamond ATR unit (Bruker Optics, Milan, Italy). Number of scans *n* = 25.
- **Degradation kinetics:** The degradation test was performed both in phosphate buffer saline (PBS) and in collagenase aqueous solution. Lyophilized scaffolds were weighted, placed into a Petri dish containing 4 mL of PBS (0.1 M; pH 7.5–8.0) or 0.01 mg/mL collagenase solution (Collagenase Enzyme type I from *Clostridium histolyticum*), and kept at 37 °C. At specific time points (1, 3, 7, 10 days for PBS and 6, 24, 48, 168 h for collagenase solution), samples were washed five times with milli-Q water to eliminate salt residues, then frozen at − 80 °C, lyophilized, and weighted to determine the percentage of degradation. Experiments were repeated four times.
- **Swelling properties:** Each dry scaffold was photographed using a digital camera and subsequently placed in a Petri dish containing 4 mL of distilled water at 37 °C for 4 h. After this period, scaffolds were photographed again and percentage variations of thickness and area were measured using the software ImageJ. In addition, water uptake was determined by evaluating the percentage change in weight of the scaffolds before and after 4 h of swelling in distilled water at 37 °C. For each type of scaffold (PHNQ-collagen, collagen, collagen UV cross-linked), five replicates were tested.
- **Mechanical tests:** Scaffolds (*n* = 5) underwent compressive test after conditioning in a climatic chamber (37 °C and 80% relative humidity, RH). Collagen-based scaffolds were used as control to compare both PHNQ-collagen composite scaffolds and UV-cross-linked collagen scaffolds. Two consecutive compression cycles were performed using a dynamometer (mod. Z005, Zwick Roell, Ulm, Germany) fitted with a 100 N load cell and connected to two plates (base plate: 150-mm diameter; compression plate: 30-mm diameter) placed at a distance of 22 mm apart. Each compression cycle accounted for a maximum deformation of the sample of 2 mm, at a speed of 2 mm s^−1^. A force–time profile was recorded. Resistance to compressive stress (expressed as maximum compressive force in N) and elastic recovery (expressed as kPa) were elaborated by TestXpert V10.11 Master software.
- **PHNQrelease kinetics:** PHNQ release kinetics were evaluated in three different conditions/solutions: distilled water, 0.9% NaCl, and 0.1 mg/mL collagenase solution. PHNQ-collagen scaffolds were immersed in 0.5 mL of the corresponding solution and kept at 37 °C. At different time points (1, 6, 24, 168 h), leachates were analyzed using the same UPLC method previously described for PHNQ detection. Experiments were performed three times using three samples.
- **ABTS assay:** ABTS (2,2′-azino-bis(3-ethylbenzothiazoline-6-sulfonic acid)) assay was performed according to Loganayaki et al. (Loganayaki et al. (Loganayaki et al. 2013) in order to measure the radical scavenging activity of PHNQ extract and 3D scaffolds. A 7 mM ABTS aqueous solution was prepared. Then, ABTS radical cation was produced by reacting ABTS aqueous solution with 2.45 mM ammonium persulfate (final concentration) and allowing the mixture to stand in the dark at room temperature overnight. Then, the solution was diluted in ethanol (about 1:75 v/v) to give an absorbance at 734 nm of about 0.7. For the PHNQ pigments, methanolic extract solutions at different concentrations were reacted with the ABTS radical solution. The same procedure was carried out using different concentrations of Trolox^®^ solutions. For the scaffolds, different sample weights were used and then reacted with ABTS radical solution. In addition, ABTS assay was performed to evaluate the antioxidant activity at different time points (1, 6, 24, 168 h) of scaffold leachate submerged in 4 mL of distilled water. Blank solution did not contain any sample. Reaction mixtures were kept in the dark for 1 h and then the absorbance was measured at 734 nm. A graph was built plotting the PHNQ concentration vs. the percentage of ABTS^•+^ remaining in solution, calculated as follows:
  $${\% }{{{ABTS}}^{\bullet +}}_{\text{remaining}} =\frac{{A}_{734\text{ nm}, 1\text{h},\text{ sample}}}{{A}_{734\text{ nm}, 1\text{h},{ blank}}}{\%}$$

### Computational Approach

- **Tripeptide sequence retrieval and analysis:** Five collagen sequences from *Paracentrotus lividus* were downloaded from the NCBI protein database (accessions: CAA61929.1, CAH10072.1, CAH10073.1, AAA29438.1, AAA29440.1) and analyzed in R using functions for sequence manipulation from the seqinr package (Charif 2007). The frequency of all possible tripeptides was calculated by dividing the number of occurrences of each triplet by the total number of triplets in each sequence, and then taking the average. We then focused on the most frequent tripeptides centered around arginine (R) or glutamic acid (E). The obtained results are reported in the Supplementary Information.
- **Molecular dynamics calculations:** Theoretical calculations aimed at a better understanding of the nature of the intermolecular interactions between collagen and PHNQs have been performed adopting the semiempirical tight binding-based quantum chemistry (QC) method GFN2-xTB in the framework of molecular dynamics (MD), in order to explore the wide variety of possible conformer ensembles at reduced computational effort, with respect to more rigorous ab initio methods.

After a set of preliminary tests (see Supplementary Information) on a selection of the most abundant amino acids found in *P. lividus* sea urchin collagen, namely arginine, aspartic acid, glutamic acid, and hydroxyproline, we opted for the GRD tripeptide as collagen representative. Glutamic acid and hydroxyproline were eventually discarded because, according to our simulations, they did not form any significant interaction with PHNQs. When running dynamics for complex systems which include different molecules non-covalently bound one to each other, it is possible to encounter dissociation of the global system in the dynamics, with molecules moving away instead of interacting: in order to avoid this, we have confined the simulation in a sphere by a repulsive potential, making feasible the study of the bound complex. The computational analyses have been run for 100 ps at a temperature of 300 K. The GRD tripeptide has been forced to stay in an elongated configuration in order to better mimic the fibrils of collagen. The interaction with Spinochrome A (SpA) and Spinochrome B (SpB) molecules has been treated separately, starting in both cases from a sandwich GRD-Spino-GRD with GRD parallel one to each other and placed at a distance of 9 Å. The presence of water as solvent has been considered explicitly including in the input files a shell of 54 water molecules free to move inside the simulation box, obtaining a total number of atoms equal to 281 and 276 for the sandwiches GRD-SpA-GRD and GRD-SpB-GRD, respectively. Full details of the computational approach can be found in the Supplementary Information.

### Cell Viability Assay

- **Cell culture:** Normal human dermal fibroblasts (NHDF) were cultured in Dulbecco’s Modified Essential Medium (DMEM; Sigma Merck, Darmstadt, Germany) high glucose (4.5 g L^−1^) supplemented with 20% (*w/v*) fetal bovine serum (FBS) (Sigma Merck, Darmstadt, Germany), 2 mM L-glutamine (Sigma Merck, Darmstadt, Germany), and a mixture of 100 U mL^−1^ penicillin and 100 mg mL^−1^ streptomycin (Sigma Merck, Darmstadt, Germany). Cells were cultured in a humified 5% (*v/v*) CO_2_ incubator at 37 °C. Cells were refreshed every 2–3 days and were passaged at sub-confluence (80–90%). For the experiments, cells were used at passages 6–7.
- **Compound preparation:** Collagen was suspended in sterile ultrapure water to a final concentration of 1 mg mL^−1^ while PHNQs were reconstituted in Hybri-Max sterile-filtered dimethyl sulfoxide (DMSO; Sigma Merck, Darmstadt, Germany) at a final concentration of 10 mg mL^−1^ (the stock solution was kept in the dark due to compound photosensitivity). Furthermore, a PHNQ-collagen mixture solution was prepared with a final collagen concentration of 10 mg mL^−1^ and PHNQ concentration of 1 mg mL^−1^ (*i.e*., PHNQs/coll = 10% w/w). All the so obtained stock suspensions were subsequently diluted in culture medium to final concentrations (see below) for in vitro testing. The final concentration of vehicle (DMSO) solution never exceeded the 1% (*v/v*).
- **Cell viability assay:** The effect of collagen and PHNQs alone or in combination on cell viability was assessed by the 3-(4,5-dimethylthiazol-2-yl)−3,5-diphenyltriazolium bromide (MTT) assay (Sigma Merck, Darmstadt, Germany) according to ISO 10993–5 (International Organization for Standardization, 2009). NHDF cells were seeded in a 96-well microplate at a final density of 10^4^ cells/well. After 24 h, cells were exposed in triplicate to increasing concentrations of collagen (100–1000 μg mL^−1^), PHNQs (1–100 μg mL^−1^), and their mixture (collagen:PHNQs, 1:10–1:10^6^ dilution in culture medium) for 24 h. Eventually, 10 μL of MTT solution was added to each well (5 mg mL^−1^ in PBS) and incubated for 1 h in the dark at 37 °C and 5% CO_2_. The reaction was stopped by the addition of 20 μL of lysis solution (20% (w/v) sodium dodecyl sulfate and 50% (v/v) dimethylformamide at pH 4.7). Plates were read for OD at *λ* = 590 using a multilabel plate reader (Victor™ X4 2030, PerkinElmer, Waltham, MA, USA). Cells exposed to vehicle were used as control. The experiment was repeated at least three times.

### Statistical Analyses

Considering the low number of samples, the non-parametric Kruskal–Wallis’ test and Dunn’s multiple comparison test were used to evaluate the significance of the differences among the degradation kinetics (*n* = 4, physiological conditions), hydration grades (*n* = 5), and water uptake (*n* = 5) results. The non-parametric Kruskal–Wallis’ test, Dunn’s (*n* = 4), and Mann–Whitney (*n* = 2) statistical tests were used to evaluate the significance of the differences among the degradation kinetics (enzymatic conditions) results. The non-parametric Kruskal–Wallis’ test was used to evaluate the significance of the differences among and mechanical tests (*n* = 5).

All the above data are expressed as mean ± standard deviation.

For cell viability assays, groups were compared to assess statistical differences using the one-way ANOVA test with a Bonferroni “post hoc” test. Cell viability assay data are expressed as mean ± standard error of the mean. Differences were considered statistically significant when *p* < 0.05. Analyses were performed using GraphPad Prism software version 9 (San Diego, CA, USA).

## Results

### Amino Acid Characterization of Sea Urchin Collagen

After having verified the collagen purity (Figure S1), the amino acid composition of collagen extracted from *P. lividu*s was analyzed following hydrolysis and derivatization and it is reported in Table 1. The amino acid composition of the sea urchin collagen sample reveals a typical collagen-like profile, with particularly high concentrations of glycine, proline, hydroxyproline, and glutamic acid, corresponding to 20.89, 13.03, 8.06, and 13.21 g per 100 g of collagen, respectively. Additionally a relevant proportion of methionine was also detected. On the other hand, *P. lividus* collagen displays a low amount of phenyl alanine and lysine (0.74 and 0.86 g/100 g, respectively).

**Table 1:** Summary table comparing the amino acid composition of *P. lividus* collagen with that of other common sources including human (bone; Eastoe 1955), bovine (achille’s tendon; Gauza-Włodarczyk et al. 2017), and fish (skin; Gauza-Włodarczyk et al. 2017). Dashes (–-) indicate the absence or non-measurement of a particular amino acid. Results are expressed in g/100 g

**Amino acids**	**Sea urchin** (*P. lividus*)	**Human**	**Bovine**	**Fish**
Hydroxyproline	8.06	14.01	8.15	5.68
Aspartic acid	6.57	6.7	7.49	6.64
Threonine	3.74	2.35	2.19	2.84
Serine	6.15	4.06	3.39	3.44
Glutamic acid	13.21	11.4	11.53	10.61
Proline	13.03	15.3	13.92	13.55
Glycine	20.89	25.8	19.22	21.83
Alanine	6.65	10.9	9.51	10.54
Valine	2.50	2.97	2.45	2.27
Methionine	2.73	0.84	0.71	2.25
Isoleucine	1.33	1.88	1.87	1.57
Leucine	2.96	3.6	3.67	2.95
Tyrosine	1.45	0.86	2.02	1.32
Phenylalanine	0.74	2.49	4.38	3.03
Hydroxylysine	–-	0.62	–	–
Lysine	0.86	4.40	3.11	3.38
Histidine	0.79	0.96	2.25	1.21
Arginine	8.35	8.8	7.30	7.56

### Morphological and Biochemical Analyses of Collagen-PHNQ Biomaterials

For the purpose of this work, PHNQs were extracted from sea urchin waste according to (Marzorati et al. 2021). The composition of the extract, as determined by UPLC-PDA, confirmed the presence of Spinochrome A (67.7 ± 5.6%) and Spinochrome B (32.3 ± 2.5%), with a final PHNQ yield of 0.07 ± 0.01% (w/w) over the initial dry biomass weight.

**Fig. 1:**
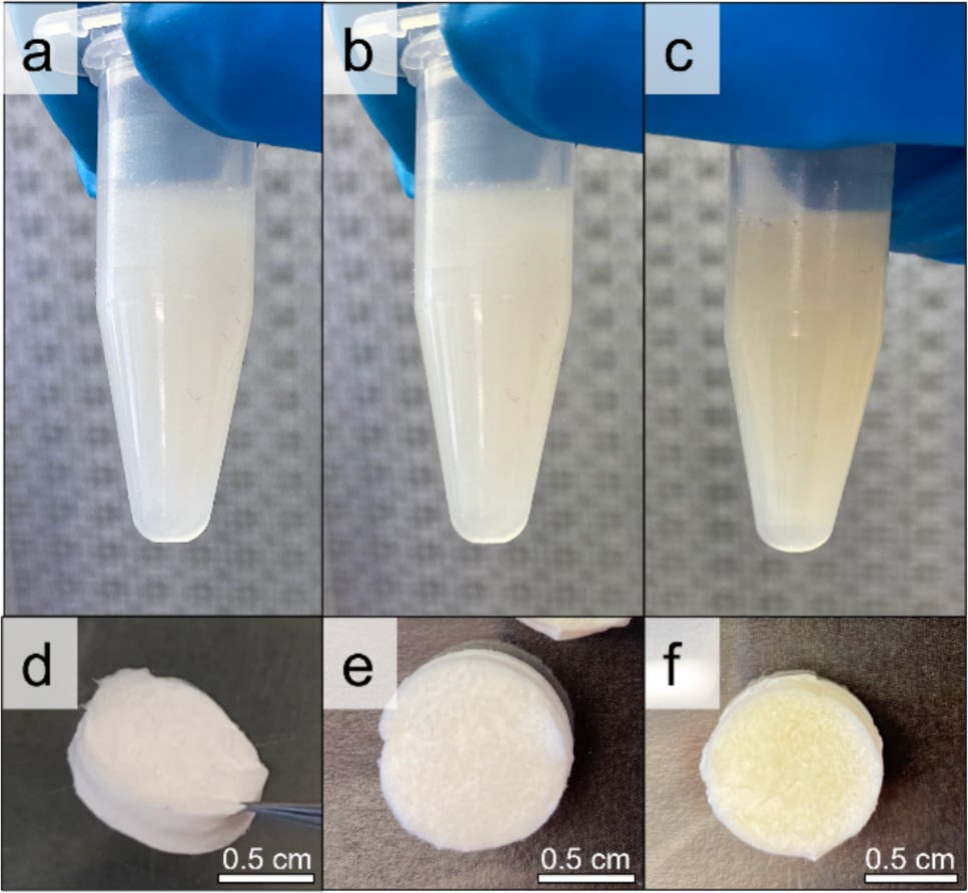
Collagen suspensions of: **a**) control (0% PHNQ in collagen), **b**) 1% PHNQ in collagen, and **c**) 10% PHNQ in collagen. Scaffolds of: **d**) collagen, **e**) collagen-1%PHNQ, and **f**) collagen-10%PHNQ

PHNQs were added to the collagen suspension in different percentages in the range from 1 to 50% w/w. At 1% and 10% (Fig. 1b, c), a homogeneous suspension, similar to the control (0%, Fig. 1a), was obtained. At 50% w/w, consistent macroaggregates (picture not shown) were formed and separated from the solution, indicating a destabilization of the collagen suspension, and thus hindering the subsequent scaffold production.

**Fig. 2:**
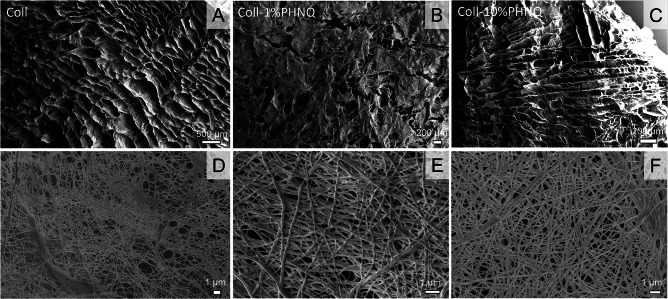
SEM images of **A**–**D** collagen-based scaffold (coll), **B–E** collagen-based scaffold added with 1% PHNQ (coll-1%PHNQ), and **C**–**F** collagen-based scaffold added with 10% PHNQ (coll-10%PHNQ), used for porosity measurements

Therefore, only the control (0%) and the 1% and 10% PHNQ-loaded suspensions were used to produce the corresponding lyophilized scaffolds (Fig. 1 d, e, and f), hereafter referred as collagen, collagen-1%PHNQ, and collagen-10%PHNQ. The 1% and 10% preparation (collagen-1%PHNQ and collagen-10%PHNQ) were then investigated for their structural features by scanning electron microscopy (SEM) to understand any potential difference in comparison to control scaffold made of sole collagen (Fig. 2). The ultrastructure of both the collagen-1%PHNQ scaffold and collagen-10%PHNQ scaffold was similar to the control scaffold, highlighting the absence of any artifacts or aggregates.

In all the three types of samples, the native collagen fibrils were in fact homogeneously dispersed in a tight network, as already described for sea urchin collagen scaffolds (Ferrario et al. 2020). This suggests that PHNQs are well dispersed in the collagenous matrix and do not interfere with the scaffold production.

The presence of PHNQ was found not to affect the fibrils diameter, calculated from SEM pictures as (a) 201 ± 79 nm, (b) 277 ± 65, and (c) 243 ± 59 with no differences among the three samples. The mean pore size of collagen, collagen-1%PHNQ, and collagen-10%PHNQ scaffolds was 204 ± 61, 227 ± 82, and 181 ± 41, respectively (as calculated from SEM images). No statistical differences were observed among the three samples (Kruskal–Wallis + Dunn’s test).

Given the absence of any morphological difference, 10% PHNQ loading was selected as the optimal level for all the subsequent analyses, aiming at maximizing and detecting any further added-value brought by the presence of PHNQs.

**Fig. 3:**
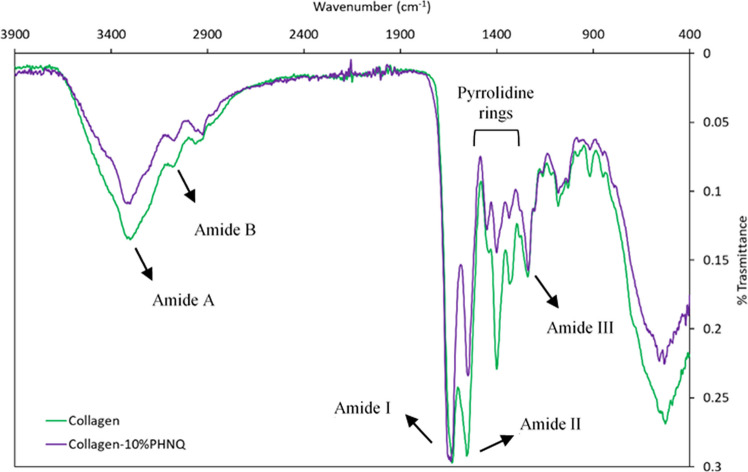
FT-IR spectrum of sea urchin collagen and collagen-10%PHNQ

FT-IR spectroscopy of pure collagen scaffold was conducted to investigate the protein secondary structure. Figure 3 shows the IR spectra of pure collagen and collagen-10%PHNQ scaffolds in the frequency range 3900–400 cm^−1^. As expected, all the scaffolds exhibit the characteristic main absorption bands of collagen type I. The absorption band at *υ* = 3300 cm^−1^ is attributed to the amide A N–H stretching vibrations. The N–H stretching vibration of amide B was found at *υ* = 3075 cm^−1^. The band of the collagen fingerprint at *υ* = 1630 cm^−1^ is typical of amide I, showing the peptide secondary structure and hydrogen bonding between N–H and C = O, generated by stretching vibration of C = O in polypeptide backbone of the protein. The amide II band was found at *υ* = 1555 cm^−1^. The peaks identified in the region between *υ* = 1440 cm^−1^ and *υ* = 1320 cm^−1^ correspond to the stereochemistry of the pyrrolidine rings of the amino acid proline and hydroxyproline (Júnior et al. 2015). The band at *υ* = 1239 cm^−1^ corresponds to amide III due to in-plane C-N stretching and N–H in plane bending vibrations from amide linkages.

When comparing the scaffold types (collagen vs. collagen-10%PHNQ), no main differences could be observed in terms of band frequencies, major variations being detectable only in the relative peak height values, indicating a certain hindrance in the vibrations attributable to the presence of PHNQ.

### Structural Stability

**Fig. 4:**
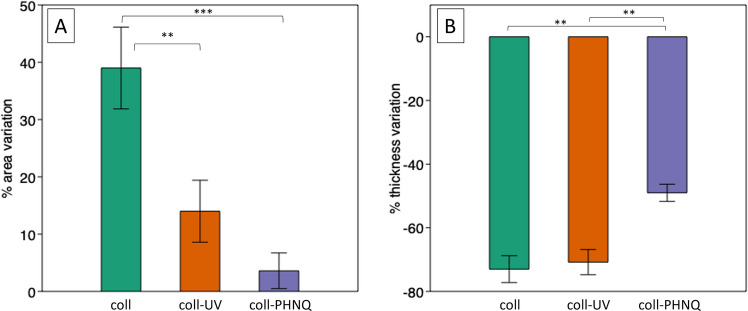
Bar plots showing in **a** the percentage of area variation (vertical axis) and in **b** the percentage of thickness variation (vertical axis) after 3h hydration for controls (coll), controls stabilized by UV (coll-UV), and composites (coll-PHNQ) (horizontal axis); errors bars show the standard error, ***p < 0.001, **p < 0.01 (Dunn’s test). Statistical analysis results are reported in Supplementary Information (Table S1)

The structural stability of all the scaffolds was tested in terms of shape (area and thickness) variation after 3 h of full hydration, in order to mimic a wound environment. The observed changes (%) are shown in Fig. 4. For the sake of a deeper understanding, composite scaffold (labeled in the figure as coll-PHNQ) was compared both to pure collagen scaffold (coll) and collagen cross-linked scaffold (coll-UV), obtained in the absence of PHNQ but subjected to a UV-light treatment to induce cross-linking. All the scaffolds displayed an increase in terms of % of area variation, due to hydration. However, the increase was significantly lower in case of composites scaffold (coll-PHNQ: 1.52%; *p* < 0.001, Dunn’s test) and controls stabilized by physical cross-linking (coll-UV: 14.19%; *p* < 0.01, Dunn’s test) compared to controls (coll: 37.75%). A similar behavior was obtained for the thickness variation, displayed in Fig. 4b. All the scaffolds collapsed after hydration, but the thickness reduction was significantly lower for the composite (coll-PHNQ: − 48.61%) compared to both controls (coll: − 73.10%; *p* < 0.01, Dunn’s test) and UV-stabilized controls (coll-UV: − 70%; *p* < 0.001, Dunn’s test).

**Fig. 5:**
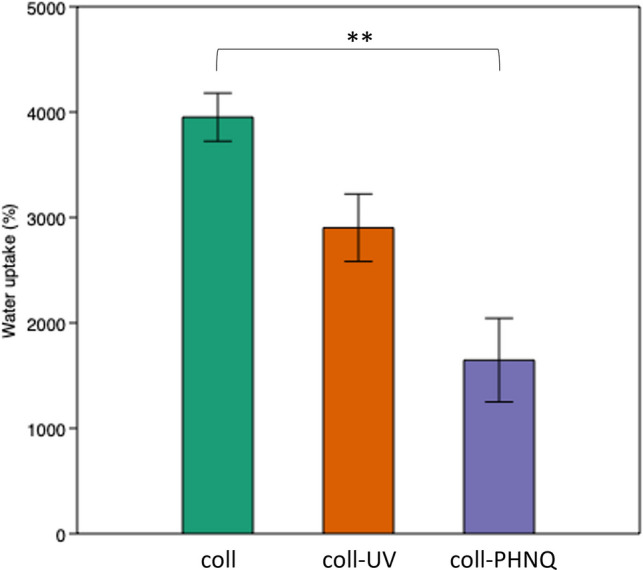
Bar plot showing the water uptake (expressed as increase (%) in weight) for controls (coll), controls stabilized by UV (coll-UV), and composites (coll-PHNQ) following hydration; errors bars show the standard error, **p < 0.01 (Dunn’s test). Statistical analysis results are reported in Supplementary Information (see Table S2)

Measurements, as displayed in Fig. 5, indicate a significant water uptake for all scaffolds always higher than 1000% (coll: 3952%; coll-UV: 3273%; coll-PHNQ: 1647%, *p* < 0.01; Kruskal–Wallis test).

Furthermore, data follow a specific trend: composite scaffold is the one displaying the lowest uptake (1647%), confirming its higher stability, suggesting a more compact configuration.

**Fig. 6:**
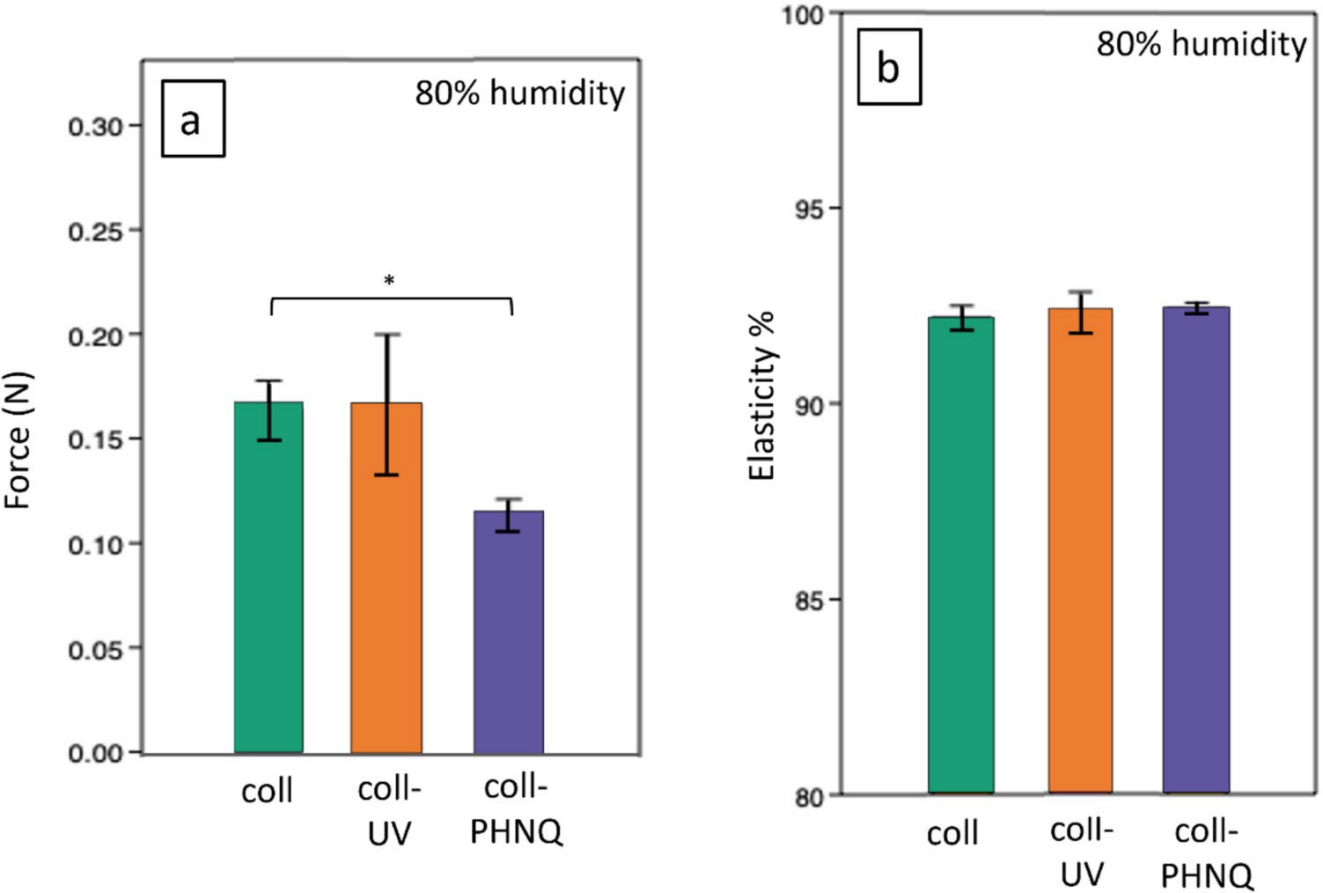
**a**) Scaffold stiffness (mean ± st. dev) expressed in force (N) under 80% humidity. Error bars show the standard deviation, *p < 0.05 (Kruskal–Wallis + Dunn’s test). **b**) Scaffold elastic recovery (mean % ± st. dev) expressed in percentage under 80% humidity. Statistical analysis results are reported in Supplementary Information (see Table S3)

Mean resistance to compressive stress (stiffness) of the scaffolds ranged from 0.1137 to 0.1735 N (Fig. 6a). While there was no significant difference between coll and coll-UV and between coll-UV and coll-PHNQ, a statistical difference was found between coll and coll-PHNQ (Kruskal–Wallis + Dunn’s test, *p* = 0.0162). The composite scaffolds, contrarily to the stability results shown when subjected to hydration, appear slightly weaker in terms of compressive stress resistance, even though this difference was not detected in terms of elastic recovery. In fact, elastic recovery (Fig. 6b), expressed in kPa, ranged from 1.5 to 5.7 kPa and was higher than 90% in all samples. Both compressive stress and elastic recovery results are in line with literature data on biomaterials for skin regeneration (Yang et al. 2018).

### Degradation Kinetics

The degradation kinetics of the fabricated biomaterials was compared to the one of Integra^®^, a commercial reference benchmark, made of bovine collagen (Dantzer et al. 2003; Jeng et al. 2007).

**Fig. 7:**
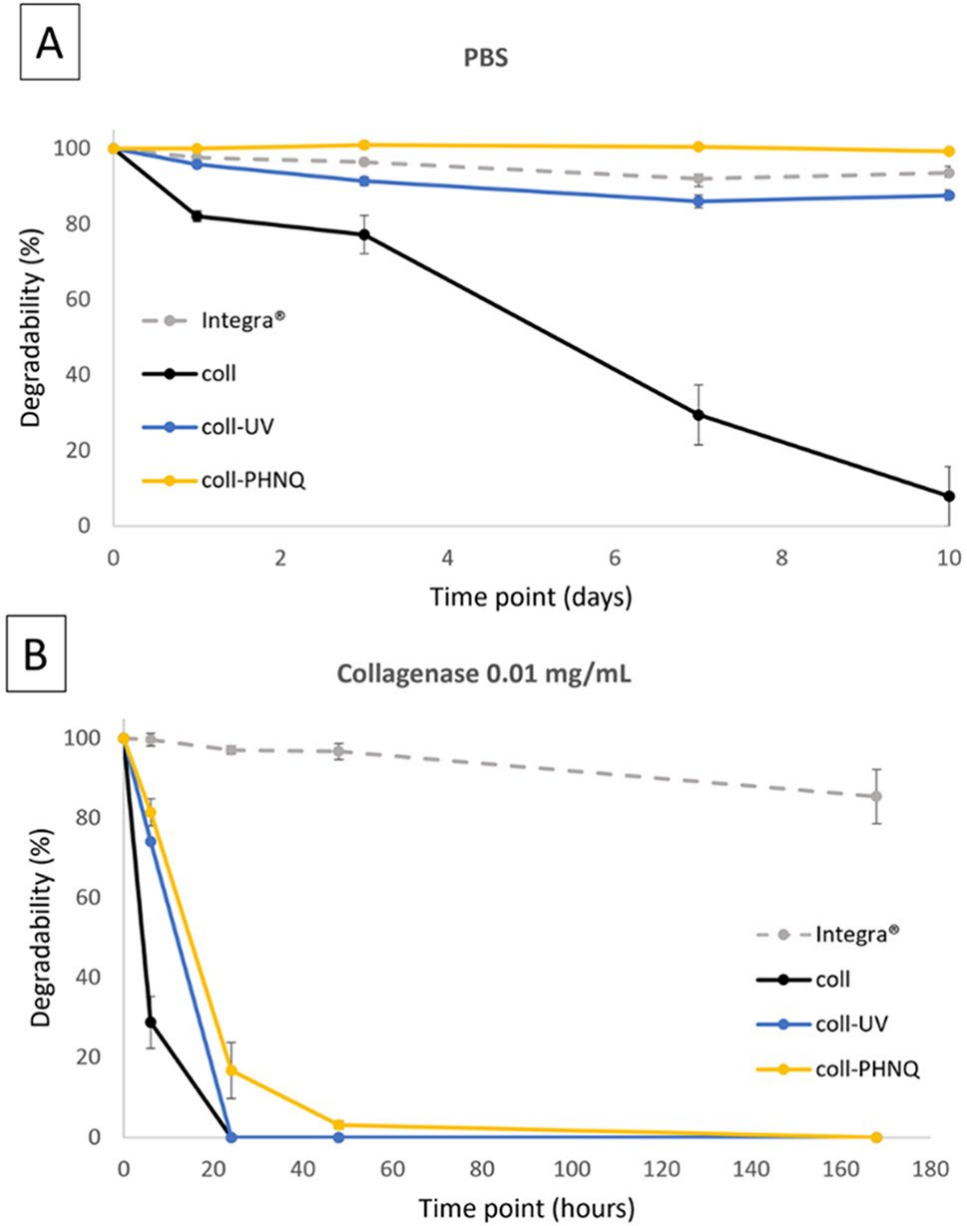
**a**) Degradation curves in PBS for the different types of scaffolds: Integra^®^, collagen (coll), collagen stabilized by UV (coll-UV), and composites (coll-PHNQ). Dots represent means ± standard error (bars). Statistical analysis results (Kruskal–Wallis and Dunn’s test) are reported in Supplementary Information (Table S2). **b**) Degradation curves in collagenase solution for the different types of scaffolds: Integra^®^, collagen (coll), collagen stabilized by UV (coll-UV), and composites (coll-PHNQ). Dots represent means ± standard error (bars). Statistical analysis results (Kruskal–Wallis, Dunn’s test, and Mann–Whitney test) are reported in Supplementary Information (Table S3)

The degradation curves in PBS (Fig. 7a) showed that composites display a trend similar to UV cross-linked-collagen scaffolds and Integra^®^, with the following loss of biomaterial masses after 10 days: 0.68% (coll-PHNQ), 12.35% (coll-UV), and 6.4% (Integra®). Differently, simple collagen scaffolds degraded significantly faster than all the other biomaterials, losing up to 90% (92.11%: coll) of the initial mass within 10 days. These data further confirm that the presence of PHNQs is able to stabilize the collagen matrix, delaying the degradation process. In collagenase solution (Fig. 7b), a more challenging environment for collagen-based biomaterials, all the scaffolds made of sea urchin collagen (both controls and composites) showed a significantly faster degradation kinetics compared to Integra^®^. Indeed, after only 48 h of enzymatic treatment, the formers were almost completely degraded whereas Integra^®^ still maintained about 97% of its original mass. Even after 1 week in collagenase, less than 20% of the biomaterial was degraded. Nevertheless, among the sea urchin-derived biomaterials, the composite coll-PHNQ was apparently the most resistant to collagenase-induced degradation, at least within the first 24 h, with a residual mass corresponding to about 17%, compared to the 0% remaining mass of both controls and UV-cross-linked controls.

### PHNQ Release Kinetics

**Fig. 8:**
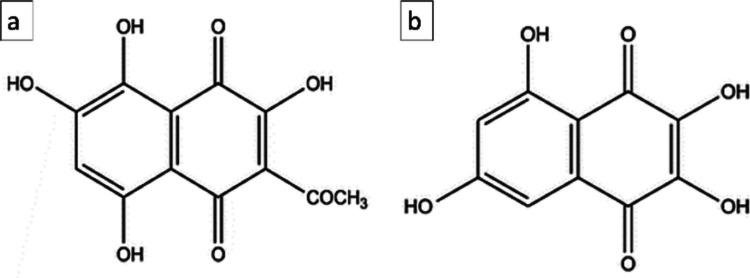
Chemical structures of SpA (**a**) and SpB (**b**)

From the results of a previous work (Marzorati et al. 2021), it was found that two main different PHNQ compounds are present in the extract: Spinochrome A (SpA) and Spinochrome B (SpB), whose chemical structures are displayed in Fig. 8.

The PHNQ release kinetics from the scaffold was evaluated analyzing the leachates in different aqueous solutions: distilled water, 0.9% NaCl, and 0.1 mg/mL collagenase solution. However, ultrahigh performance liquid chromatography (UPLC) clearly indicates that nor SpA neither SpB were detectable in any of the leachate solutions at any considered time points (1, 6, 24, 168 h) at retention times corresponding to SpA and SpB.

### Atomistic View of the Cross-linking Process

In order to provide a possible explanation for why the presence of PHNQs within the collagen fiber network produces positive effects on the scaffold’s stability suggesting a more compact configuration, tight binding molecular dynamics methods (Bannwarth et al. 2019) were used to study the interaction of PHNQs with the tripeptide glycine-arginine-aspartic acid (GRD hereinafter), selected as collagen representative. This choice represents a compromise between heavier ab initio simulations and lighter force field approaches, considering the large number of atoms involved and is also motivated by chemical intuition, since the three selected amino acids are between the most abundant in the *P. lividus* collagen (see Table 1) and are characterized by the presence of free functional groups which are crucial for the establishment of hydrogen bonding with PHNQs, favoring a better and stronger cross-linking, if any.

**Fig. 9:**
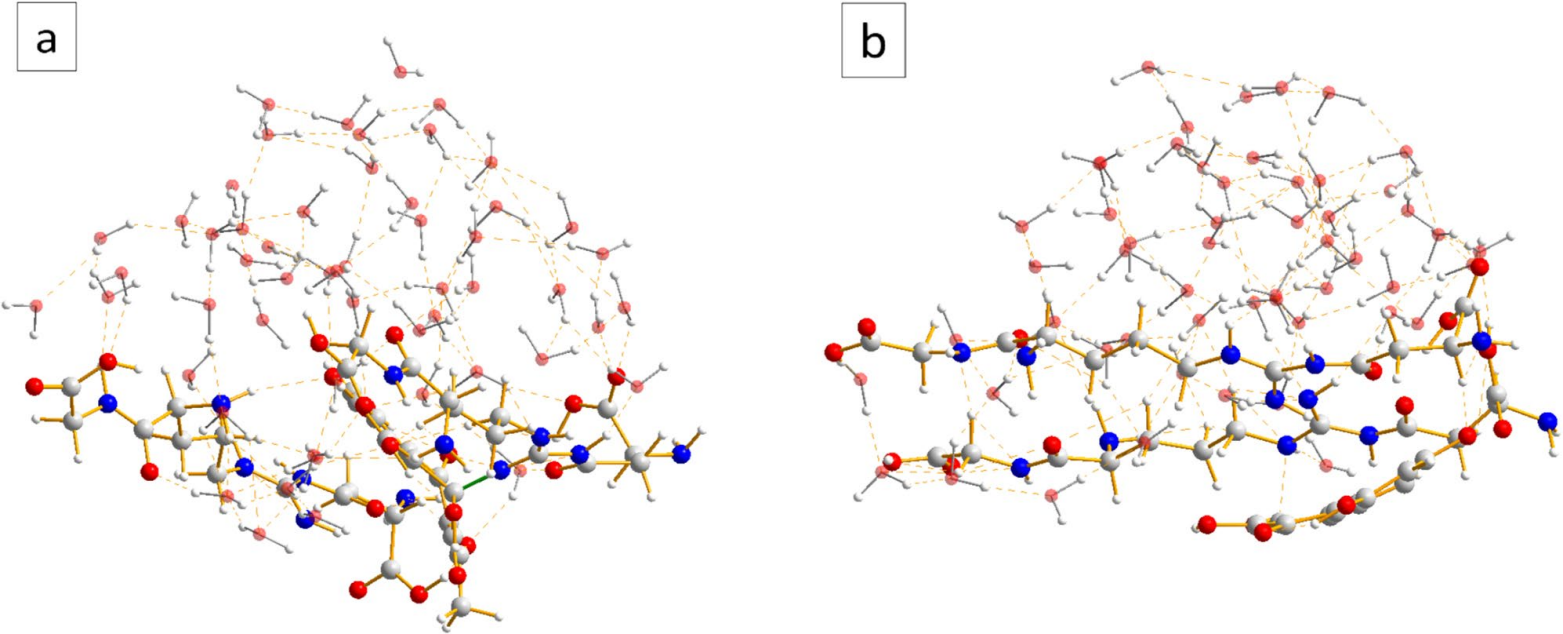
Example of conformation of the: **a**) GRD-SpA-GRD and **b**) GRD-SpB-GRD adduct during the MD simulations (after thermalization). HB interactions are highlighted with dashed orange lines. The green stick flags the new covalent bond formed between the collagen representative and Spinochrome A

In the course of the 100 ps of MD simulation, it was found that SpA can directly bind to GRD through a covalent N–C bond (N–C bond distance = 1.474 Å). In Fig. 9, it can be seen that this bond (panel **a**), as well as the intermolecular hydrogen bonds (HBs) occurring between GRD and SpB (panel **b**), competes with the intermolecular contacts between the tripeptide and water molecules, relegating the latter on one side of the organic adduct. For more details, see Supplementary Information.

**Fig. 10:**
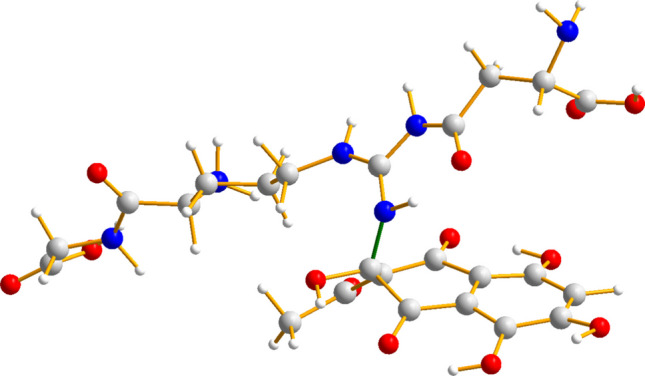
Adduct between the collagen fragment GRD and SpA. The green bond is the new covalent bond formed during the MD simulation

The preferred binding site for the GRD tripeptide is one of the terminal N atoms of the arginine fragment, which forms a covalent bond with one of the aromatic carbon atom of Spinochrome A, as shown in Fig. 10. This bond is likely the signature of the formation of a permanent cross-link.

### Evaluation of the Antioxidant Activity: ABTS Assay

The ABTS assay was utilized to compare the radical scavenging activity of the freshly extracted PHNQ solution, re-solubilized lyophilized PHNQ extract (serving as a control to verify that the lyophilization process did not alter the antioxidant properties of PHNQs), dry collagen scaffold, and coll-PHNQ composite scaffold. Trolox^®^, a well-known antioxidant molecule (Anthony and Saleh 2013; Iqbal et al. 2015), was employed as a reference standard for antioxidant activity. The obtained EC_50_ values, detailed in Table 2, corroborated the notable antioxidant activity of the pristine extracted PHNQs, corresponding to 0.022 ± 0.007 mg/mL. Subsequently, the antioxidant activity of PHNQs after lyophilization was evaluated, resulting in an EC_50_ of 0.036 ± 0.004 mg/mL, slightly higher than that of the PHNQ extract, indicating an only slightly lower antioxidant activity, demonstrating that the antioxidant activity of PHNQs was largely preserved after lyophilization and re-solubilization. Next, the leached solution from the pure collagen scaffold (coll) was assessed for its antioxidant activity, revealing an EC_50_ of 0.8 ± 0.1 mg/mL, one order of magnitude higher than that of the other samples. This confirmed that the eventual released peptides, arising from the collagen scaffold degradation, did not exhibit significantly high antioxidant activity. Finally, the ABTS assay was conducted directly on the solid composite scaffold (coll-PHNQ) to examine whether the biocomposite material maintained a similar antioxidant activity to that of PHNQs in solution. The obtained EC_50_ value confirmed that the antioxidant activity was retained on the composite scaffolds, yielding an EC_50_ of 0.06 ± 0.03 mg/mL, corresponding to the same order of magnitude as the lyophilized PHNQs.

**Table 2:** EC_50_ (mg/mL) values for Trolox^®^, PHNQ extract, lyophilized PHNQ extract, biomaterial made of sole sea urchin collagen, and composite biomaterial (PHNQ-collagen)

Samples	EC_50_ (mg/mL)
Trolox®	0.0035 ± 0.0005
PHNQ extract	0.022 ± 0.007
Lyophilized PHNQ extract	0.036 ± 0.004
Collagen	0.8 ± 0.1
PHNQ-collagen	0.06 ± 0.03

### Cell Viability Assay (MTT)

**Fig. 11:**
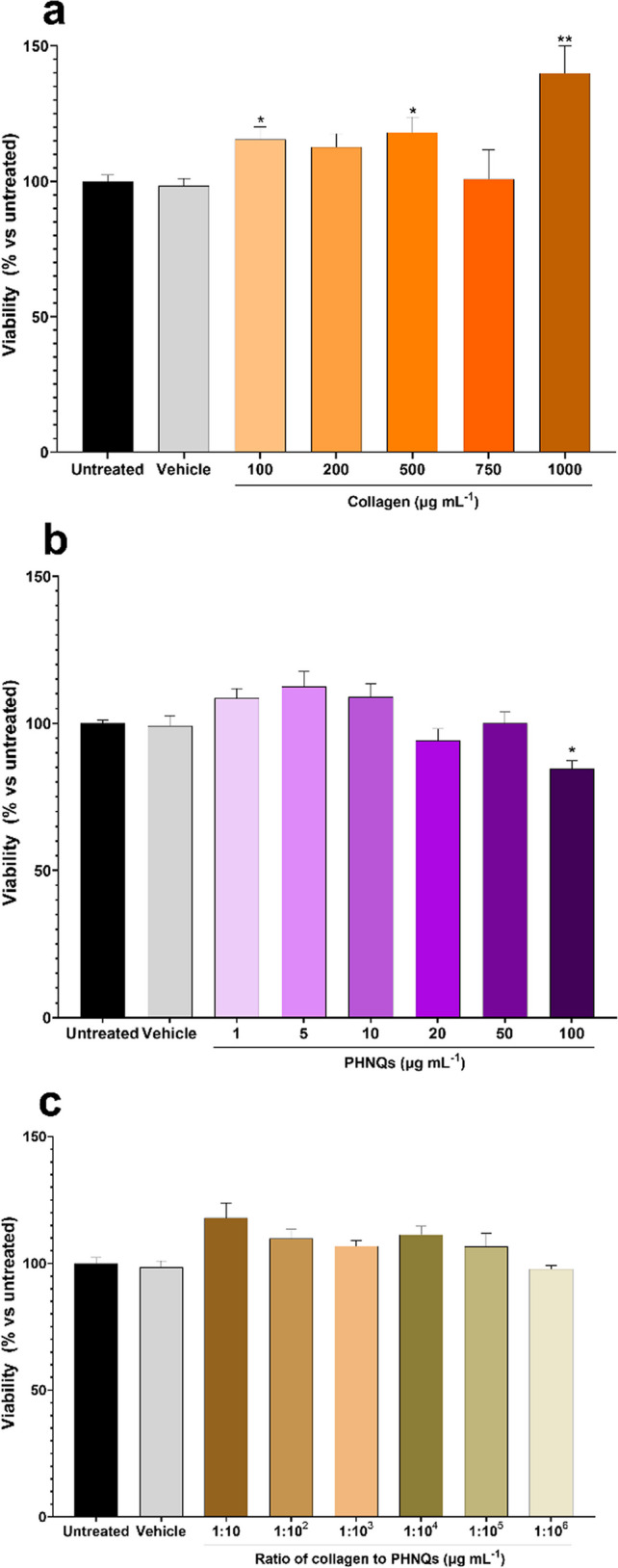
Cell viability of NDHF exposed to different concentrations of: **a**) sea urchin collagen, **b**) PHNQs, or **c**) a combination of both. Statistical differences are expressed as “vs. vehicle”; *p < 0.05, **p < 0.01

Cells exposed to collagen showed a slight increase in percentage of metabolically active cells respect to untreated samples, as displayed in Fig. 11, especially at 100, 500, and 1000 μg mL^−1^ who showed a statistically significant difference compared to the sole vehicle. On the other hand, PHNQs showed toxicity at high concentrations (100 μg mL^−1^). Nonetheless, when NHDF were exposed to the combination of the two compounds, cell viability was not significantly affected. Moreover, as shown in Figure S10 in the representative microphotographs of NHDF exposed to different concentrations of PHNQs (1, 10, 20, 100 μg mL^−1^), the presence of PHNQs did not directly affect the morphology of cells.

## Discussion

The present study was addressed to achieve a complete valorization of sea urchin’ food waste toward a highly functional biomaterial for future biomedical applications. The whole sea urchin waste was profitably used to extract biologically relevant molecules, such as native fibrillar collagen and potent antioxidant compounds (PHNQs) and combined them to fabricate a “clean”, sustainable and multifunctional biomaterial. The success of this approach depends on ensuring that the biomaterial meets specific criteria—such as appropriate structure, composition, degradation rate, and cell viability—that are essential for its functionality.

Given that the backbone of the produced biomaterials is based on collagen, a preliminary amino acid analysis was necessary in order to understand its composition and, more importantly, to better study its interaction when in contact with PHNQ.

The amino acid composition of collagen from *P. lividus*, compared with that of other organisms used as sources of collagen, is presented in Table 1.

Notably, *P. lividus* exhibits amino acid values were rather similar to those of human and mammalian collagen (Eastoe 1955; Gauza-Włodarczyk et al. 2017), with some exceptions (Table1). This includes lower glycine and hydroxyproline (the two collagen-typical structural amino acid) content in sea urchin than in human collagen, possibly suggesting slight differences in overall structural organization of collagen (Piez and Gross 1960). Other differences are particularly notable for methionine and glutamic acid, notably higher in* P. lividus* collagen. It is relevant to note that a higher methionine content can be found in collagen from aquatic sources (both sea urchin and fish), thus suggesting potential environment-related differences. Methionine is an essential amino acid which plays roles in mammalian metabolism including DNA methylation (Cavuoto and Fenech 2012). Methionine is employed in pharmaceutical formulations for treating infections and is recommended as a dietary supplement for addressing disorders arising from insufficient protein intake. Glutamic acid is important for various metabolic processes, including amino acid synthesis and energy production; for this reason, it has been used also in biomaterial development, particularly loaded in collagen-base composite scaffold (Sanapalli et al. 2022) All these features underscore the potential utility of this sea urchin species as a sustainable and alternative collagen source for several applications, including both pharmacological or nutraceutical formulations. Its amino acidic composition can be also a useful feature in biomaterials for tissue regeneration, as the collagen scaffold is expected to be progressively hydrolyzed during wound healing, thus locally providing a source of important amino acids. Indeed, the distinctive composition of* P. lividus* collagen may confer specific advantages in terms of bioactivity, solubility, and structural properties (Amarowicz et al. 2012; Paola et al. 2019).

When observing the morphology (SEM imaging) of PHNQ-added scaffolds in comparison to simple collagen-based ones, no differences were detected. In all cases, fibril diameter resulted similar to that of mammalian ones (Gelse et al. 2003) and to marine collagen fibril sizes (25–300 nm), as already reported in the literature (Di Benedetto et al. 2014; Ferrario et al. 2017). Scaffolds pore size was in line with previous results, highlighting a heterogeneous porous shape and size throughout the full thickness, in the presence of both lamellar and network structures. Mean porosity appears suitable to allow cell seeding and infiltration (Loh and Choong 2013).

In terms of optimization of PHNQ loading, different percentages of polyphenolic PHNQs were tested to determine the maximum amount that could be loaded onto the composite biomaterial. PHNQs were added to the collagen suspension at increasing concentrations: 1%, 10%, and 50% w/w. However, concentrations exceeding 10% resulted in a destabilization of the collagen suspension and formation of consistent insoluble aggregates. Given this result, 10% loading was selected as the optimal PHNQ percentage over collagen, taking into account several aspects simultaneously: (i) it allowed a homogeneous scaffold to be produced, (ii) it was loaded with a sufficiently high amount of PHNQs to be potentially detectable (higher than instrumental limits of detection) in the subsequent release kinetics tests, and (iii) it is cytocompatible, even assuming total release of PHNQs in the culture medium, taking into account previous in vitro tests (Melotti et al. 2023).

We then evaluated the structural stability of the produced scaffolds under wet conditions, *i.e.,* resembling the in vivo environment. To this purpose, swelling and water uptake measurements were compared across the different scaffold types: collagen (coll), collagen stabilized by UV (coll-UV), and composites (coll-PHNQ). More specifically, an increase in surface area was observed across all scaffold types (coll: 38%, coll-UV: 14%, coll-PHNQ: 2%); however, those containing PHNQs exhibited the lowest reduction and therefore a better structural stability. Similarly, while all scaffolds collapsed (*i.e.*, reduced their thickness) to some extent after hydration (coll: − 73%; coll-UV: − 70%, coll-PHNQ: − 49%), the composite scaffolds experienced significantly lower thickness reduction, again suggesting enhanced structural stability possibly due to the formation of bonds with collagen fibrils. This result was also confirmed by FT-IR analysis, evidencing an increased rigidity of the scaffold in the presence of PHNQ, with a reduction of vibrational possibilities. Data from water uptake test are in line with these results. In fact, all the collagen scaffolds showed high water uptake, this partially due to the nature of the extracted sea urchin collagen, which is naturally enriched in surface glycosaminoglycans (GAGs) (Di Benedetto et al. 2014). Indeed, full maintenance of surface GAGs likely contributes to this high hydrophilicity and water retention capability (Yeh and Lin 2008). This water uptake is indicative of scaffold responsiveness to aqueous environments, such as blood, with materials exhibiting high water holding capacity correlating positively with improved angiogenesis, beneficial for tissue regeneration (Zhu et al. 2023). Nevertheless, composite scaffolds (coll-PHNQ) demonstrated a significantly lower water uptake. All together, these results suggest that the presence of PHNQs apparently stabilized the scaffold, even more efficiently than the physical cross-linking with UV treatment. A possible explanation is that the “cross-linking” exerted by PHNQ made the overall structure of the coll-PHNQ scaffold more cohesive and therefore more restricted to structural deformations (swelling) and water uptake.

Scaffolds were also tested for mechanical properties at 80% humidity (at 37 °C) to mimic a wound environment. Stiffness results show that composite scaffolds (coll-PHNQ), as well as collagen-based scaffolds, both cross-linked and non-cross-linked (coll and coll-UV), are in line with the literature (human skin estimated stiffness ranges from 0.06 to 0.86 N/mm^2^) (Graham et al. 2019). Composite scaffolds (coll-PHNQ) were statistically different from collagen-based scaffolds (coll): despite the increased chemical resistance of coll-PHNQ, due to the presence of polyphenols, the stiffness was slightly lower than that of collagen-based scaffolds (coll). Since the lower water uptake of coll-PHNQ suggests a more cohesive structure, a higher stiffness would have been expected. This apparent discrepancy may be due to the chemical interactions introduced by PHNQ. By covalently binding to collagen, PHNQ can interfere with fibrillar packing and disrupt non-covalent interactions that normally support mechanical strength, reducing the ability of the material to withstand compression by affecting fibril alignment and load bearing capacity. Finally, all tested scaffolds showed elastic recovery of more than 90%, which is comparable to the average value of human skin elasticity (Yang et al. 2018).

Aiming at further mimicking the tissue healing physiological conditions in vivo, since these biomaterials naturally degrade as new tissue forms in wounds, degradation tests were performed. The degradation kinetics of scaffolds in contact with humid environments were assessed in PBS (as a mild physiological condition) and collagenase (as an enzymatic treatment mimicking proteases present in human tissues) solutions. Results revealed similar degradation profiles in physiological conditions for composites (coll-PHNQ) and collagen physically stabilized by UV-light (coll-UV), comparable to the commercial benchmark, Integra^®^, a bovine collagen-based scaffold enriched in glycosaminoglycans (GAGs) and chemically cross-linked with glutaraldehyde (Taupin et al. 2023), exhibiting minimal degradation even after 10 days. Integra^®^ was chosen as a reference due to its established clinical use in tissue regeneration (Dantzer et al. 2003; Jeng et al. 2007; Taupin et al. 2023). On the other side, collagen scaffolds (coll) completely degraded within 10 days. The behavior of collagen stabilized by UV scaffolds (coll-UV) depended on collagenous fibril interactions during cross-linking, while composite scaffolds (coll-PHNQ) behavior points to a further evidence of collagen-PHNQ interaction and stabilization. Degradation observed in collagenase solutions confirmed the slow degradation for Integra^®^, likely due to its chemical cross-linking and additional stabilizing components, such as GAGs. Conversely, composites (coll-PHNQ) exhibited a behavior more similar to the other collagen-based scaffolds, anyway still slower than collagen stabilized by UV scaffolds (coll-UV) and collagen scaffolds (coll), completely degraded within 24 h. This evidence again suggests that the presence of PHNQs hinders collagenase access, supporting the potential of composite scaffolds in wound healing, where scaffold degradation rates must match tissue formation rates (Nokoorani et al. 2021).

Given the experimental evidence of specific interactions between PHNQ and collagen, the release kinetics of PHNQs from composite scaffolds was then evaluated in distilled water, 0.9% NaCl, and collagenase enzyme solutions (0.1 mg/mL). It was indeed confirmed the absence of any release of PHNQs in any solution, indicating strong bonds formed between collagen (or eventual released collagenous peptides, arising from the collagen scaffold degradation) and PHNQs. From the last results, it appears clear that PHNQ-collagen biomaterials should not be considered as drug (antioxidants) delivery systems. It was then fundamental to assess whether any antioxidant activity could be still present on the final scaffolds. In the literature are in fact reported some case studies in which the antioxidant properties were maintained on the solid biomaterials, due to the presence of free (and not sterically hindered) functional groups responsible for radical quenching activity, the base mechanism of antioxidant properties (Serpen et al. 2007). Through ABTS assay, PHNQ-collagen composite scaffolds were confirmed to exhibit higher antioxidant activity (EC_50_ = 0.06 ± 0.03 mg/mL) compared to collagen scaffolds (EC_50_ = 0.8 ± 0.1 mg/mL), in this last case likely due to peptides with a slight antioxidant activity. These results confirmed the possibility to confer an antioxidant additional character to collagen-based scaffolds and demonstrated the feasibility of producing biocomposites, combining regenerative properties with antioxidant activity conferred by PHNQs polyphenolic compounds.

Considering the studied composites at a molecular level, it can be hypothesized that hydrogen and covalent bonds govern the cross-linking between aromatic small molecules and collagen, as for example in the case of polyphenols (Shavandi et al. 2018). Our computational analysis suggests a possible mechanism for the cross-linking process, providing an explanation for both the stabilization of collagen and its augmented resistance to degradation in biological systems. The cross-linking ability is higher for Spinochrome A, while for Spinochrome B, the intermolecular interactions between the PHNQ and the selected collagen-representative include only intermolecular HBs.

The picture offered by the MD simulations show that water molecules prefer to establish a network of intermolecular HBs between themselves, rather than interpose between the organic molecules in the composite mixture. This provides a possible mechanism at molecular level for the experimental observation about the hydrophobicity of the collagen fiber and the resulting lower water uptake of the coll-PHNQ scaffold.

Lastly, in order to assess the biocompatibility of the PHNQ-collagen mixture, an in vitro cell viability test was performed. Dermal fibroblasts (NHDF) were exposed to different concentrations of collagen and PHNQs, alone or in combination. As previously mentioned, sea urchin collagen has been widely used as a beneficial (bio) material for proliferation and culture of different kind of cells (Di Benedetto et al. 2014; Ferrario et al. 2017) in these experiments, we corroborate this observation as cells showed an increase of viable cells when exposed to sea urchin-derived collagen. However, as expected, high doses of PHNQs (100 mg mL^−1^) alone showed a toxic effect on cell viability (Song et al. 2010) suggesting cytocompatibility for doses < 100 µg mL^−1^. Concomitantly, when cells were exposed to the combination of PHNQ and collagen the cytotoxic action of the pigments was not observed, even for PHNQ nominal concentrations of 100 µg mL^−1^. This further supports the idea that PHNQs are sequestered/cross-linked to collagen fibrils and are not therefore bioavailable for cells. Overall, these data suggest that the combination of collagen with PHNQs might be not harmful for cells or tissues, thus making composite biomaterials suitable for biomedical application.

## Conclusions

In this study, a successful, effective, and clean transformation of sea urchin waste, sourced from restaurants and sea urchin processing companies, was carried out toward the development of high-value marine-derived product. This process aligns with the principles of the circular economy and promotes the growth of the blue bioeconomy. The development of collagen-based composite scaffolds incorporating polyhydroxynaphthoquinones (PHNQs) derived from sea urchin waste presents a promising strategy for enhancing wound healing outcomes. Results suggested a strong interaction between collagen and PHNQs. Tight binding molecular dynamics simulations supported the experimental evidence of improved structural stability in the composite biomaterials and showed no detectable release of PHNQs over time. In particular, it was found that, in presence of water as a solvent, a new covalent bond can be formed between the glycine-arginine-aspartic acid tripeptide, selected as collagen representative, and one PHNQ, hence providing a microscopic explanation of the experimental evidences. Furthermore, the exposure of human dermal fibroblasts to different concentration ratios of collagen-PHNQ composites further demonstrates that the combination of these compounds do not affect cell viability, thus demonstrating the cytocompatibility of the developed biomaterials.

Future research should focus on further optimizing the composition and properties of these composite scaffolds, as well as investigating NDHF behavior when directly seeded onto the 3D biomaterials to observe how it might affect cell viability and morphology to further assess a potential clinical application and their regenerative efficacy.

## Supplementary Information

Below is the link to the electronic supplementary material.

ESM 1 (DOCX 2.46 MB)

## Data Availability

All data generated or analysed during this study are included in this published article and its supplementary information files.
