# Supplementary material for: Seas of Renewal: Turning Sea Urchin Waste into Polyhydroxynaphtoquinone-Collagen Biomaterials for Regenerative Medicine
Source: Mar Biotechnol (NY). 2025 Aug 30;27(5):131. doi: 10.1007/s10126-025-10504-2 (PMC12398438; doi:10.1007/s10126-025-10504-2)
Supplement: Supplementary file 1 — (DOCX 2.46 MB) [file 10126_2025_10504_MOESM1_ESM.docx]

**Seas of Renewal: Turning Sea Urchin Waste into Polyhydroxynaphtoquinone-Collagen Functional Scaffolds for Regenerative Medicine**

Giordana Martinelli,^a^ Stefania Marzorati,^a^* Margherita Roncoroni,^a^ Luciano Magro,^b^ Matteo Brilli,^c^ Giangiacomo Beretta,^a^ Luca Melotti,^d^ Anna Carolo,^d^ Giulia Zivelonghi,^d^ Marco Patruno,^d^ Raffaella Soave,^e^ Mario Italo Trioni,^e^ and Michela Sugni^a^

1. Department of Environmental Science and Policy, Università degli Studi di Milano, Via Celoria 2, 20133, Milano, Italy.
2. Department of Agronomy, Food, Natural Resources, Animals and Environment, University of Padua, Viale dell’Università, 16, 35020, Legnaro (Padova), Italy.
3. Department of Biosciences, Università degli Studi di Milano, Via Celoria 26, 20133, Milano, Italy.
4. Department of Comparative Biomedicine and Food Science, University of Padua, 35020, Legnaro (Padova), Italy.
5. National Research Council of Italy, Institute of Chemical Science and Technologies “Giulio Natta”, via Golgi, 19, 20133, Milan, Italy.

* Corresponding author: stefania.marzorati@unimi.it.

**
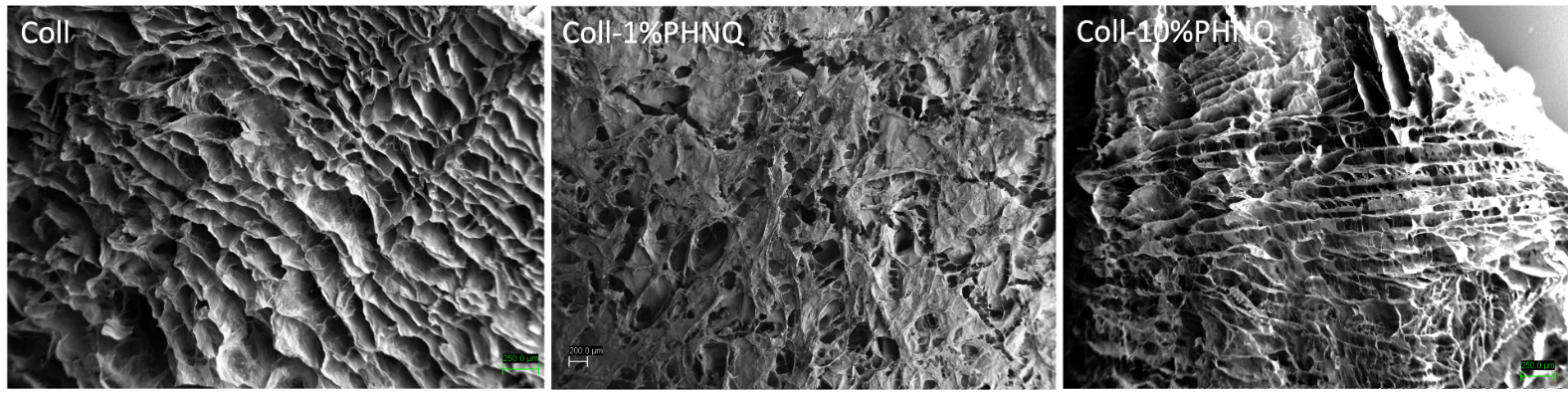
**

**Figure S1.** SEM images of collagen based scaffold (coll), collagen based scaffold added with 1% PHNQ (coll-1%PHNQ) and collagen based scaffold added with 10% PHNQ (coll-10%PHNQ), used for porosity measurements.

**Table S1.** Kruskal Wallis and Dunn’s test relevant to **Figure 4**. The statistical data results speak for a significant difference for p<0.05, p<0.005 and p<0.001.

| **Scaffolds** | **Variation %** | **p value** |
| --- | --- | --- |
| coll - coll10%PHNQ | Area | 0.0018 |
| coll - collUV | Area | 0.0282 |
| coll - coll10%PHNQ | Thickness | 0.0048 |
| collUV - coll10%PHNQ | Thickness | 0.0113 |

**Table S2.** Kruskal Wallis and Dunn’s test for each time point relevant to Figure 5. The statistical data results speak for a significant difference for p<0.05, p<0.005 and p<0.001.

| **Scaffolds** | **Time point (days)** | **P** |
| --- | --- | --- |
| Integra® - coll | 1 | 0.0329 |
| coll – coll10%PHNQ | 1 | 0.0005 |
| Integra® - coll | 3 | 0.0170 |
| collUV – coll10%PHNQ | 3 | 0.0170 |
| coll10%PHNQ - Integra® | 3 | 0.0003 |
| Integra®- coll | 7 | 0.0212 |
| coll - coll10%PHNQ | 7 | 0.0003 |
| collUV – coll10%PHNQ | 7 | 0.0212 |
| coll - Integra® | 10 | 0.0171 |
| coll - coll10%PHNQ | 10 | 0.0003 |
| collUV – coll10%PHNQ | 10 | 0.0171 |

**Table S3.** Kruskal Wallis, Dunn’s test and Mann-Whitney test relevant to Figure 6. The statistical data results speak for a significant difference for p<0.05, p<0.005 and p<0.001.

| **Scaffolds** | **Time point (hours)** | **p value** |
| --- | --- | --- |
| Integra^®^ - coll | 6 | 0.0003 |
| Integra^®^ - coll10%PHNQ | 24 | 0.0294 |
| Integra^®^ - coll10%PHNQ | 48 | 0.0303 |

**Full details of simulations within the Molecular Dynamics approach**

A large set of preliminary tests has been performed to find the most favorite pair constituted by an amino acid (AA) and a spinochrome. The AAs considered are arginine, aspartic acid, glutamic acid, and hydroxyproline (**Figure S1**). All of them have been simulated firstly as single AAs and then coupled with both SpA and SpB (**Figure S2**), forming different dimers.

| 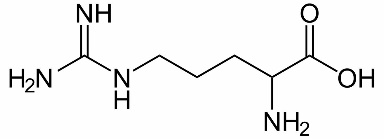 | 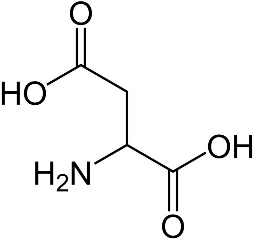 | 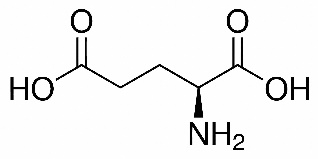 | 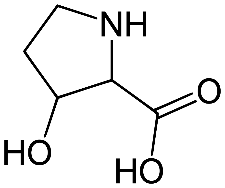 |
| --- | --- | --- | --- |
| Arginine | Aspartic acid | Glutamic acid | Hydroxyproline |

**Figure S2.** The four amino acids considered in the Molecular Dynamics simulations.


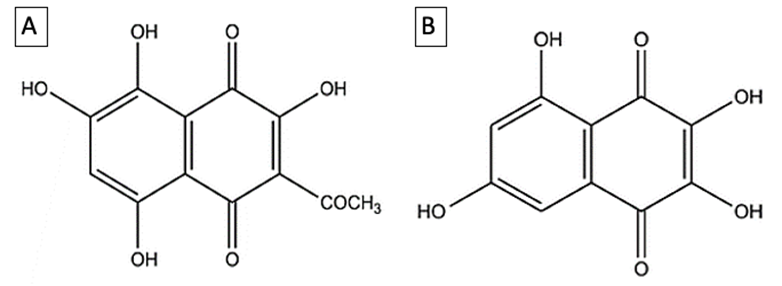


**Figure S3**. Chemical structures of SpA (A) and SpB (B).

In the molecular dynamics (MD) runs the water solvent has been considered both implicitly (polarizable continuum model-PCM in the framework of analytical linearized Poisson-Boltzmann (ALPB) Ehlert S., Stahn M., Spicher S., Grimme S. *"Robust and Efficient Implicit Solvation Model for Fast Semiempirical Methods"*. J. Chem. Theory Comput. (2021) 17, 4250-4261. doi: 10.1021/acs.jctc.1c00471) and explicitly, giving rise to very similar results.

The following graphs show the evolution of the pairs studied over a 200 picoseconds time interval. The points on the graphs are the energies of the stable (optimized) configurations obtained starting from the coordinates generated during the MD simulation. This procedure has been implemented within a in-house code (M.I.T. private communication), following the idea of the Basin Hopping algorithm (Wales David J., Doye Jonathan P. K. *"Global Optimization by Basin-Hopping and the Lowest Energy Structures of Lennard-Jones Clusters Containing up to 110 Atoms"*. J. Phys. Chem. A (1997) 101, 5111–5116).


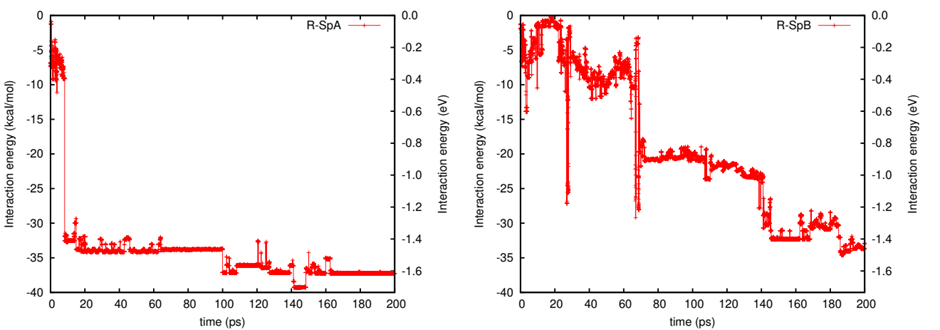


**Figure S4**. Pairs arginine-SpA (left) and arginine-SpB-arginine (right): evolution of the interaction energies during the 200 ps (horizontal axis) of the MD run.


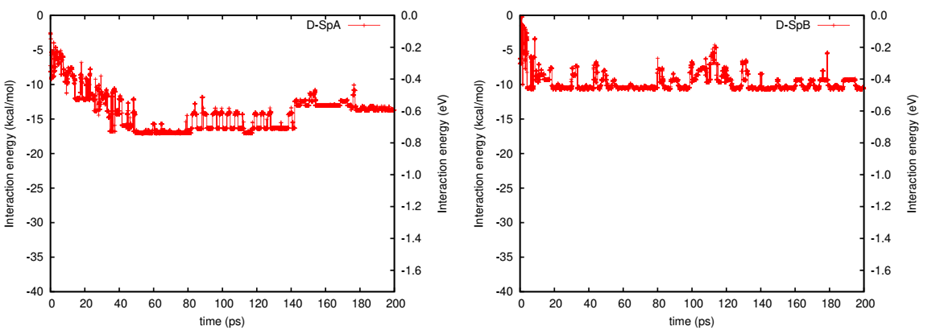


**Figure S5**. Pairs aspartic acid-SpA (left) and aspartic acid-SpB (right): evolution of the interaction energies during the 200 ps (horizontal axis) of the MD run.


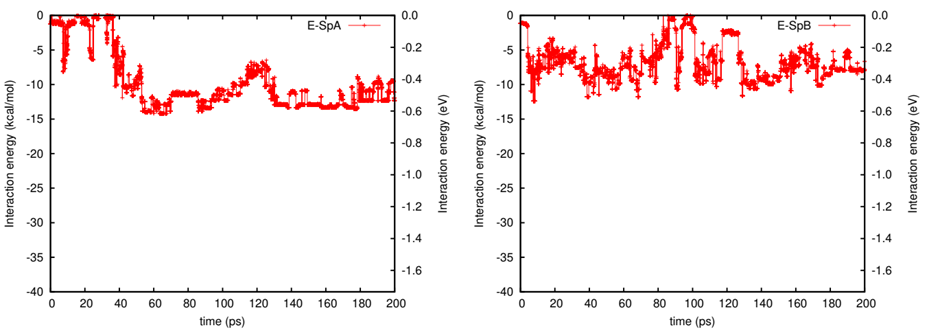


**Figure S6**. Pairs glutammic acid-SpA (left) and glutammic acid-SpB (right): evolution of the interaction energies during the 200 ps (horizontal axis) of the MD run.


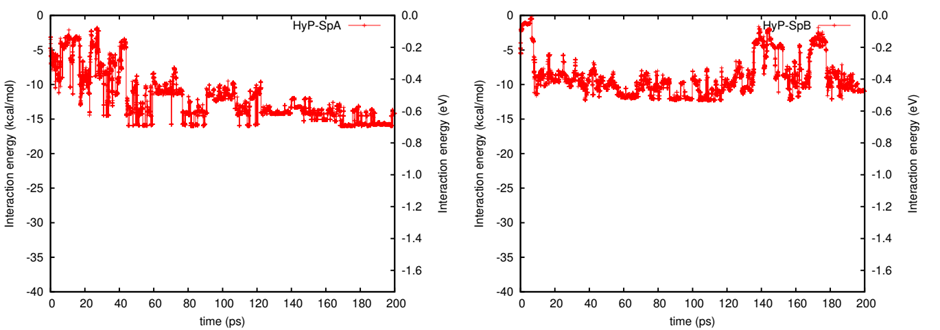


**Figure S7**. Pairs hydroxyproline-SpA (left) and hydroxyproline-SpB (right): evolution of the interaction energies during the 200 ps (horizontal axis) of the MD run.

It can be observed that the pair arginine-SpA is the only one which shows an abrupt decrease of the energy (around 8 ps of the simulation, **Figure S3**, left panel), indicating that a stable minimum is formed. Looking at the corresponding molecular configuration, we have found that this minimum is related to the formation of a covalent bond between a carbon atom of SpA and a nitrogen atom of arginine (**Figure S7**). Furthermore, once the bond is created it persists along all the simulation time considered.


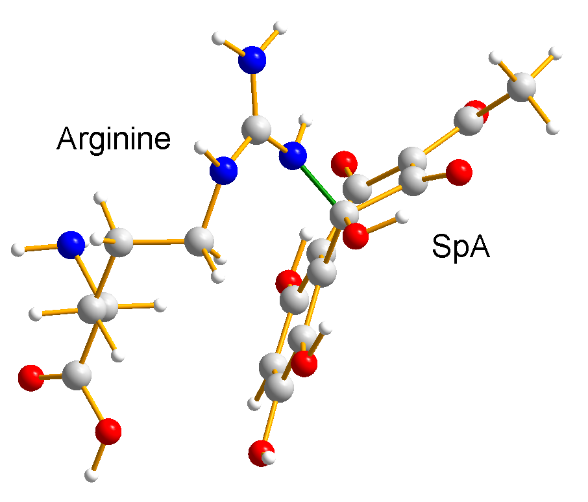


**Figure S8**. Adduct between the AA arginine and SpA. The green bond is the new covalent bond formed during the MD simulation.

In the case of the dimer arginine-SpB the interaction energy is still quite strong but, in this case, it is mainly due to the formation of a short intermolecular hydrogen bond which forms after the migration of a hydrogen atom from an hydroxyl group of SpB to and NH group of arginine (**Figure S8**).


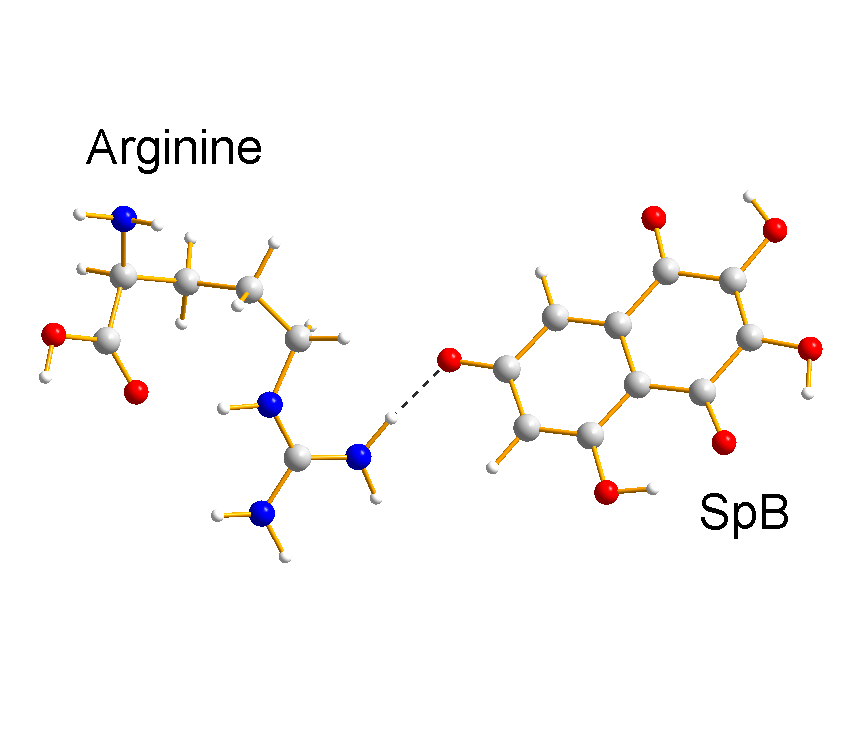


**Figure S9**. Adduct between the AA arginine and SpB. The intermolecular NH…O hydrogen bond formed during the MD simulation due to a migration of a hydrogen atom from an hydroxyl group of SpB to and NH group of arginine, is shown as dashed line.

After these preliminary tests, we moved to the study of the interaction of PHNQs with the tripeptide glycine-arginine-aspartic acid (GRD hereinafter), selected as collagen representative. This choice was motivated by intrinsic computational limits due to the large number of atoms involved, on one side, and by chemical intuition on the other. Indeed, the three selected amino acids are abundant in the *P. lividus* collagen and are characterized by the presence of free functional groups crucial for the establishment of hydrogen bonding with PHNQs, which would result in a better and stronger cross-linking, if any. The arginine AA was selected as central residue due to its strong attitude to bond to spinochromes.

Moreover, according to the reported preliminary tests, we did not consider tripeptides containing glutamic acid despite its high frequency (see **Table S1**), because of the observed weak interaction of this AA with spinochromes.

The molecular dynamic simulations have been performed using the xTB code (Bannwarth C., Ehlert S. and Grimme S., J. Chem. Theory Comput. (2019) 15, 1652–1671. doi: 10.1021/acs.jctc.8b01176) details of which are given at <https://xtb-docs.readthedocs.io/en/latest/> webpage.

The Hamiltonian is considered at the GNF2-xTB level. In the molecular dynamics runs we used the Barendsen thermostat fixed at T=300 K. The duration of the simulations has been fixed at least to 100 ps, and in all cases the thermalization has been fully reached.

Furthermore, in some cases, global optimization has been executed for each step of molecular dynamics run in order to explore many different local minima to be compared with each other.

In presence of explicit solvent (water) a consistent number of H_2_O molecules were randomly distributed around the molecules of interest to avoid any bias in the results.

**Tripeptide Sequence Retrieval and Analysis**

The frequency of all possible tripeptides was calculated with the seqinr package (…) by dividing the number of occurrences of each triplet by the total number of triplets in each sequence, and then taking the average. We then focused on the most frequent tripeptides centered around Arginine (R) or Glutamic acid (E).

**Table S4.** Frequencies of the triplets centered around Arginine (R) or Glutamate (E) considering five *P. lividus* collagen sequences as obtained through the seqinr package.

| **Triplet** | **Frequency** | **Triplet** | **Frequency** |
| --- | --- | --- | --- |
| GER | 0.0248 | GRD | 0.0162 |
| GEP | 0.0236 | TRP | 0.0107 |
| TEA | 0.0123 | LRL | 0.0093 |
| PES | 0.0107 | LRF | 0.0090 |
| LEV | 0.0093 | PRG | 0.0090 |
| EEE | 0.0090 | TRE | 0.0090 |
| FEQ | 0.0090 | GRM | 0.0086 |
| FEY | 0.0090 | ART | 0.0076 |
| IEE | 0.0090 | FRY | 0.0076 |
| KEI | 0.0090 | GRR | 0.0076 |


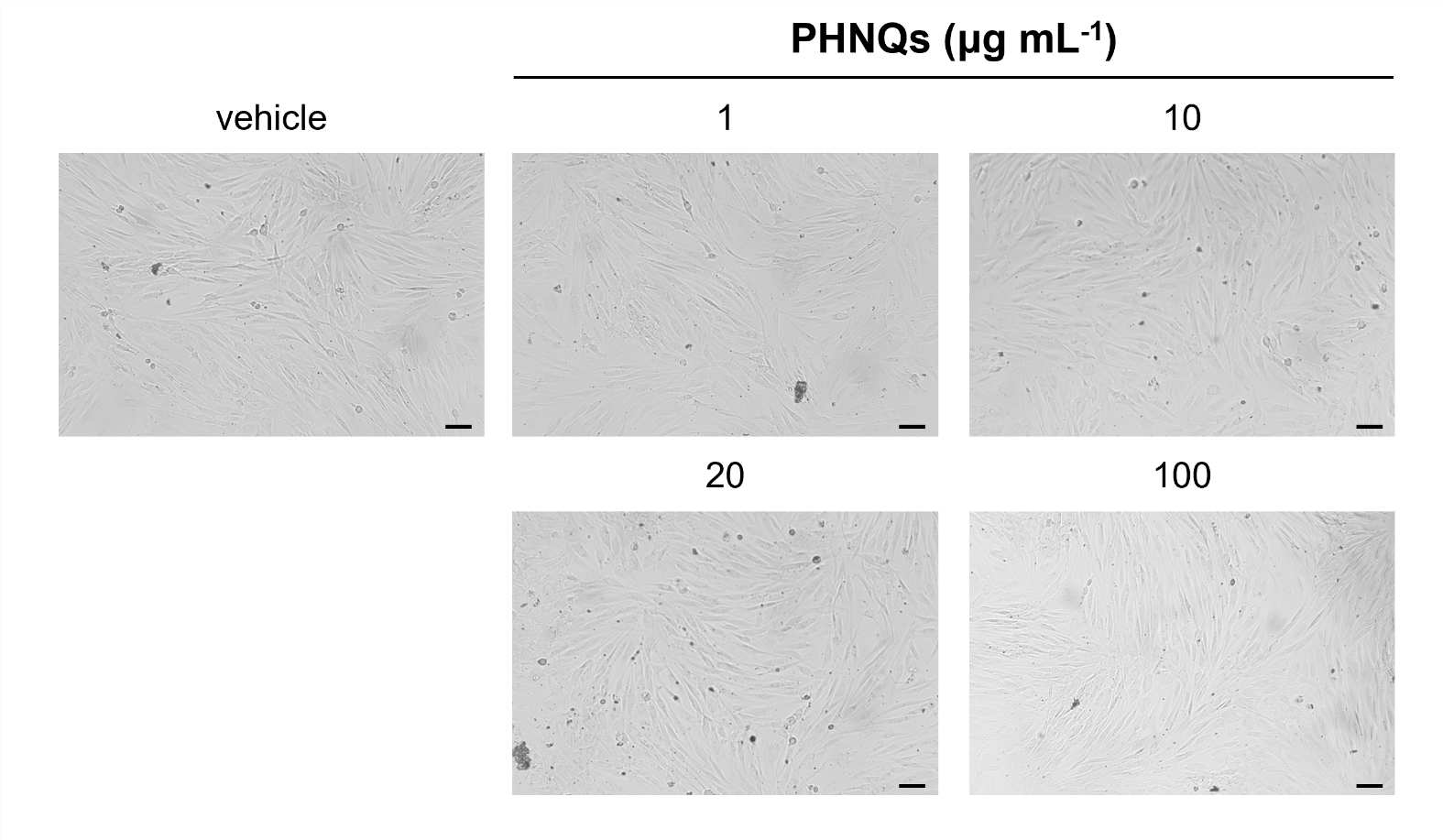


**Figure S10.** Representative image of NDHF exposed to vehicle (DMSO) or different concentrations of PHNQs (1-100 μg mL^-1^); scalebar = 10 μm.
